# Cancer-associated adipocyte promote progression and immunosuppression in triple-negative breast cancer

**DOI:** 10.3389/fonc.2026.1797152

**Published:** 2026-04-16

**Authors:** Xichao Zhang, Shenjie Zhong, Shan Liu, Xiaofeng Mu, Gang Chen, Wen Chen

**Affiliations:** 1 School of Medical Laboratory, Shandong Second Medical University, Weifang, China; 2 Department of Clinical Laboratory, Qingdao Central Hospital, University of Health and Rehabilitation Sciences, Qingdao, China; 3 School of Rehabilitation Science and Engineering, University of Health and Rehabilitation Sciences, Qingdao, China; 4 Emergency Center, Qingdao Central Hospital, University of Health and Rehabilitation Sciences, Qingdao, China

**Keywords:** cancer-associated adipocyte, epithelial-mesenchymal, transition, immunosuppressive microenvironment, triple-negative breast cancer, tumor microenvironment

## Abstract

Triple-negative breast cancer (TNBC) is characterized by highmetastatic potential and a lack of effective targeted therapies. Within the tumor microenvironment (TME) of TNBC, adipocyte can undergo transformation into cancer-associated adipocytes (CAA) through interactions with cancer cells; however, the specific role in the progress of TNBC is still not well described. This study aimed to investigate the impact of CAA on the malignant behavior of TNBC and its underlying mechanisms. CAA model was successfully established by co-culturing 3T3-L1-induced adipocytes with 4T1 cells, which exhibited characteristic features such as reduced lipid accumulation. Functional assays demonstrated that co-culture with CAA significantly enhanced the migration and invasion capabilities of 4T1 cells. *In vivo* experiments showed that co-injection of CAA with tumor cells accelerated primary tumor growth and promoted lung metastasis in mice. Mechanistic analysis revealed that in tumor tissues coexisting with CAA, E-cadherin expression was downregulated, accompanied by increased Ki67 expression and activation of the PI3K/AKT signaling pathway. Furthermore, CAA induces an immunosuppressive TME, characterized by elevated PD-L1 expression and reduced CD8^+^T cell infiltration. In conclusion, this study demonstrates that CAA promotes TNBC progression by activating epithelial-mesenchymal transition (EMT) and the PI3K/AKT pathway, as well as remodeling an immunosuppressive microenvironment, providing experimental insight into tumor-adipocyte interactions and identifying potential therapeutic targets.

## Introduction

Breast cancer is the most common malignant tumor among women worldwide. Its high heterogeneity stems not only from the molecular subtypes of cancer cells but is also closely associated with the complex TME in which it develops (1, 2). TNBC, as a distinct subtype, is characterized by the lack of expression of estrogen receptor, progesterone receptor, and human epidermal growth factor receptor 2 (HER2) (3, 4). Consequently, TNBC patients not benefit from endocrine therapy or HER2-targeted agents and have long relied primarily on radiotherapy, chemotherapy, and surgical intervention (5, 6). Although novel therapies such as immune checkpoint inhibitors have shown promise for some patients, TNBC overall remains confronted with significant challenges, including high aggressiveness, a tendency for early recurrence and metastasis, and poor prognosis (7, 8). The five-year survival rate for TNBC is notably lower than that of other subtypes, underscoring the urgent need to elucidate its progression mechanisms and explore new therapeutic strategies.

TME is an ecosystem dynamically shaped by interactions among tumor cells, immune cells, cancer-associated fibroblasts, endothelial cells, adipocytes, and other stromal components. It profoundly influences tumor initiation, immune evasion, and metastatic progression (9, 10). In breast cancer-particularly within fatty breast tissue-adipocytes constitute one of the most abundant and metabolically active cellular constituents of the TME. Recent studies have shifted from viewing adipose tissue merely as a passive energy reservoir to recognizing it as a vital endocrine and paracrine organ (9, 11, 12). When cancer cells invade and embed within the adipose stroma, they induce profound phenotypic and functional reprogramming of resident adipocytes, transforming them into CAA (13–15). Collectively, these changes foster a local microenvironment that facilitates tumor progression (16, 17).

Accumulating evidence indicates that CAA promotes tumor progression through dual mechanisms: by providing metabolic substrates, such as fatty acids, to fuel cancer cell growth, and by secreting pro-inflammatory cytokines that actively interact with tumor cells, which collectively drive cancer cell proliferation and invasion (18). However, most existing studies have regarded CAA as a relatively homogeneous pro-tumor population, focusing mainly on their direct regulation of autonomous malignant behaviors in tumor cells. The highly metastatic nature of TNBC is driven by two key synergistic processes: the EMT, which enables tumor cells to acquire migratory and invasive capabilities, and immune evasion, which allows them to circumvent immune surveillance and establish distant metastases. It remains unclear whether and how CAA can simultaneously modulate both processes, thereby driving the malignant progression of TNBC in a coordinated manner. Specifically, can CAA effectively induce EMT in TNBC cells? More critically, can CAA reshape the local immune microenvironment, for example, by upregulating immune checkpoint molecules such as PD−L1 or by influencing the infiltration and function of CD8^+^ T cells, thereby facilitating immune escape by tumor cells? To date, systematic experimental evidence and mechanistic insights into the regulatory role of CAA in the “EMT-immune suppressive microenvironment” axis in TNBC are still lacking.

This study aims to investigate the contributions of CAA to TNBC progression in two dimensions: the induction of intrinsic alterations in tumor cell properties and the remodeling of the immune microenvironment. We propose a central hypothesis: CAA synergistically promotes TNBC invasion and distant metastasis by concurrently activating the EMT process in tumor cells and fostering an immunosuppressive TME. To test this hypothesis, we will employ an integrated approach utilizing *in vitro* co-culture models, *in vivo* orthotopic tumor growth and metastasis models, and multi-level histomolecular analyses. The specific objectives are: (1) to confirm the enhancing effect of CAA on the migratory and invasive capacities of TNBC cells; (2) to evaluate the promotional role of CAA in tumor growth and pulmonary metastasis at the organismal level; (3) to preliminarily elucidate the underlying dual mechanism linked to EMT induction and immune suppression. This study is expected to provide novel insights into the complex crosstalk between tumor cells and adipocytes in TNBC and to offer potential theoretical and experimental foundations for developing microenvironment-targeted combination strategies aimed at blocking the metastatic cascade.

## Methods

### Establishment of the orthotopic mammary tumor model

Animal experiments were approved by the Institutional Animal Care and Use Committee of the University of Health and Rehabilitation Sciences (No. 2026-4001). In this study, an *in situ* mammary carcinoma model was established using 6-week-old female BALB/c mice. 4T1 cells and CAA were mixed at a ratio of 4:1 and co-injected into the fourth mammary fat pad of the mice, with 1 × 10^6^ 4T1 cells and 2.5 × 10^5^ CAA per injection. When the tumor volume reached approximately 50–80 mm³, the mice were randomly assigned to different treatment groups. Body weight and tumor volume were measured every two days post-inoculation. Tumor and lung tissues were collected on day 14 for subsequent experimental analyses. The experimental endpoint was defined as a tumor volume exceeding 1,500 mm^3^ or the appearance of symptoms requiring humane intervention.

### Cell culture

MDA-MB-231, 3T3-L1 and 4T1 cells obtained from the laboratory’s existing cryopreserved stock. Cells were cultured in high-glucose Dulbecco’s Modified Eagle Medium (DMEM) supplemented with 10% fetal bovine serum (FBS) and 1% penicillin-streptomycin. Cells were maintained in a humidified incubator at 37 °C under 5% CO_2_. Upon reaching 80-90% confluence, they were passaged using 0.25% trypsin-EDTA. Cell morphology and growth were regularly observed to ensure consistency.

### Cell induction and differentiation

3T3-L1 preadipocytes were seeded in 6-well plates and allowed to reach 90% confluence. Subsequently, the culture medium was replaced with fresh high-glucose DMEM complete medium (supplemented with 10% FBS and 1% penicillin / streptomycin) for a 2-day contact inhibition phase. To initiate adipogenic differentiation, the medium was then changed to induction medium A, containing 20 nM insulin, 1 µM dexamethasone, and 0.5 mM 3-isobutyl-1-methylxanthine (IBMX), for 2 days. This was followed by incubation in induction medium B, which contained only 20 nM insulin, for another 2 days. Finally, cells were maintained in high-glucose DMEM complete medium for an additional 2 days to promote maturation into lipid-accumulating adipocytes. Throughout the differentiation process, cells were kept in a humidified incubator at 37 °C with 5% CO_2_, and medium was refreshed every 2 days. Morphological changes and lipid droplet formation were monitored by microscopy (19).

### Co-culture assay

To specifically elucidate the role of paracrine factors secreted by adipocytes-that is, effects independent of direct cell-cell contact, extracellular matrix remodeling, or lipid transfer-we established a non-contact co-culture system using Transwell filters. This approach allows for the independent assessment of the role of the adipocyte secretome in regulating tumor cell behavior, thereby providing fundamental insights into this single pathway prior to investigating more complex interactions *in vivo*. A non-contact co-culture system was established using 6-well Transwell plates (Corning) with polycarbonate membranes (pore size: 0.4 μm). Normal adipocytes (NA) were generated by differentiating 3T3-L1 preadipocytes as described above. Mature NA were seeded in the upper inserts at a density of 1 × 10^5^ cells per insert, and 4T1 cells (1 × 10^5^ cells per well) were plated in the lower chamber. Both chambers were maintained in a shared co-culture medium (high-glucose DMEM supplemented with 10% FBS) and at 37 °C in a humidified incubator with 5% CO_2_. Medium was replaced every 48 h. After co-culture, adipocytes in the inserts were referred to as CAA and used for subsequent assays.

### Oil red staining

Prior to staining, the culture medium was gently aspirated, and the cells were washed once with PBS. An appropriate volume of staining wash solution was then added to cover the cell monolayer for 20 s. After removing this solution, the cells were incubated with Oil Red O working solution, for 20 min at room temperature. Following incubation, the staining solution was discarded, and the cells were gently rinsed with staining wash solution for 30 s, followed by a 20 s wash with PBS. Finally, a sufficient volume of PBS was added to cover the cells, and lipid droplet staining was immediately examined and photographed under a light microscope using bright-field illumination (20).

### RT-qPCR

Total RNA was extracted from NA and CAA using the TRIzol method (Gene-Better, China). After determining the concentration and purity, reverse transcription was performed to synthesize cDNA (ReverTra Ace^®^ qPCR RT Master Mix with gDNA Remover (Code No. FSQ-301), TOYOBO, Japan). The mRNA expression levels of adiponectin and the internal control GAPDH were detected on a real-time quantitative PCR instrument using SYBR Green Master Mix (THUNDERBIRD^®^ SYBR^®^ qPCR Mix, QPS-201, TOYOBO, Japan). The primer sequences were as follows: AdipoQ forward 5’-TGTTCCTCTTAATCCTGCCCA-3’, reverse 5’-CCAACCTGCACAAG TTCCCTT-3’ and GAPDH forward 5'-GGCTGCCCAGAACATCAT-3', reverse 5'-CGGACACATTGGGGGTAG-3'.

### Cell scratch assay

Once the cells reached full confluence, a scratch was introduced across the cell monolayer using a sterile 200 μL yellow pipette tip. The tip was held perpendicular to the plate and drawn steadily along pre-marked reference lines (at least three lines had been drawn on the back of the 6-well plate with a marker prior to co-culture to ensure reproducible positioning). After creating the scratches, the original culture medium was aspirated, and the cells were gently washed twice by slowly adding PBS along the wall of each well to remove detached cells. Residual liquid was carefully removed, and the medium was replaced with serum-free medium or medium containing 2% serum to minimize proliferation-driven wound closure. The same scratch regions were imaged at 0 h and 24 h using an inverted phase contrast microscope, with incubation at 37 °C under 5% CO_2_ between time points. Migration was quantified by measuring the change in scratch width between the two time points with ImageJ.

### Cell invasion assay

*In vitro* cell invasion was assessed using Matrigel-coated Transwell chambers. Briefly, thawed Matrigel was diluted 1:8 with serum-free medium on ice. Then, 60 µL of the mixture was evenly coated onto the upper surface of the Transwell membrane (pore size: 8 µm) and allowed to polymerize at 37 °C in 5% CO_2_ incubator for 3 hours. After removing excess liquid, the coated membrane was hydrated with 100 µL of serum-free medium for 30 min. Meanwhile, cells from each group were washed with PBS, trypsinized, centrifuged, and resuspended in serum-free medium to a density of 1×10^5^ cells/mL. The lower chamber was filled with 500 µL of medium containing 10% FBS, and 200 µL of the cell suspension (2×10^4^ cells) was added to the upper chamber. The chambers were incubated at 37 °C with 5% CO_2_ for 24 h. Following incubation, the chambers were rinsed with PBS. Cells on the upper surface were removed, and those on the lower surface were fixed with 4% paraformaldehyde, stained with 0.5% crystal violet, and rinsed with distilled water. Invaded cells were counted and photographed in five random fields per membrane under a microscope, and the results were expressed as the mean number of cells per field.

### Western blot

Total protein was extracted from collected cell samples using RIPA lysis buffer supplemented with protease and phosphatase inhibitors. Protein concentration was determined using the BCA assay. Based on the quantification results, equal amounts of protein were separated by SDS-PAGE and transferred onto polyvinylidene fluoride (PVDF) membranes. The membranes were blocked with 5% bovine serum albumin (BSA) for 1.5 h at room temperature, followed by incubation with primary antibodies overnight at 4 °C. The primary antibodies used included: β-actin (1:5000, Bio-Sensor, bs-0061R), E-cadherin (1:1000, Abclonal, A20798), Vimentin (1:5000, Abclonal, A19607), AKT (1:1000, Abways, CY5561), and p-AKT (1:2000, Bioss, bs-2720R). After washing, the membranes were incubated with appropriate secondary antibodies (1:50000 HUABIO, HA1001), washed again, and finally visualized for protein detection. The signal intensity of each target protein was normalized to that of the loading control.

### Tissue sectioning experiment

Mice were euthanized by cervical dislocation, and tumor tissues as well as lung tissues were promptly harvested. Tumor tissues and portions of lung tissues were fixed in 4% paraformaldehyde. All fixed tissue samples were subsequently submitted to Wuhan Servicebio Technology Co., Ltd. for paraffin embedding and section preparation.

### Bouin’s fixed

To quantify pulmonary metastasis, mice were euthanized at the experimental endpoint and lungs were excised, gently rinsed in PBS, and fixed in Bouin’s solution for 18 h at room temperature. After fixation, lungs were transferred to 70% ethanol for storage. For the quantification of metastatic nodules, surface lesions visible on each lung lobe were counted. The lungs were then systematically photographed under consistent lighting, and the number of metastatic foci was further quantified from the images to ensure accurate and reproducible assessment.

### Statistical analysis

All statistical analyses were performed using GraphPad Prism 8.0 (GraphPad Software, Boston, MA, USA). Data are presented as mean ± SD unless otherwise indicated. *In vitro* experiments were repeated at least three times independently. For comparisons between two groups, an unpaired two-tailed Student’s t-test was used for normally distributed data; otherwise, the Mann-Whitney U test was applied. For comparisons among multiple groups, one-way ANOVA followed by Tukey’s multiple-comparisons test was used for parametric data, and the Kruskal-Wallis test followed by Dunn’s *post hoc* test was used for nonparametric data. A P-value < 0.05 was considered statistically significant.

## Results

### Construction and phenotypic validation of CAA in a TNBC co−culture model

**Figure 1:**
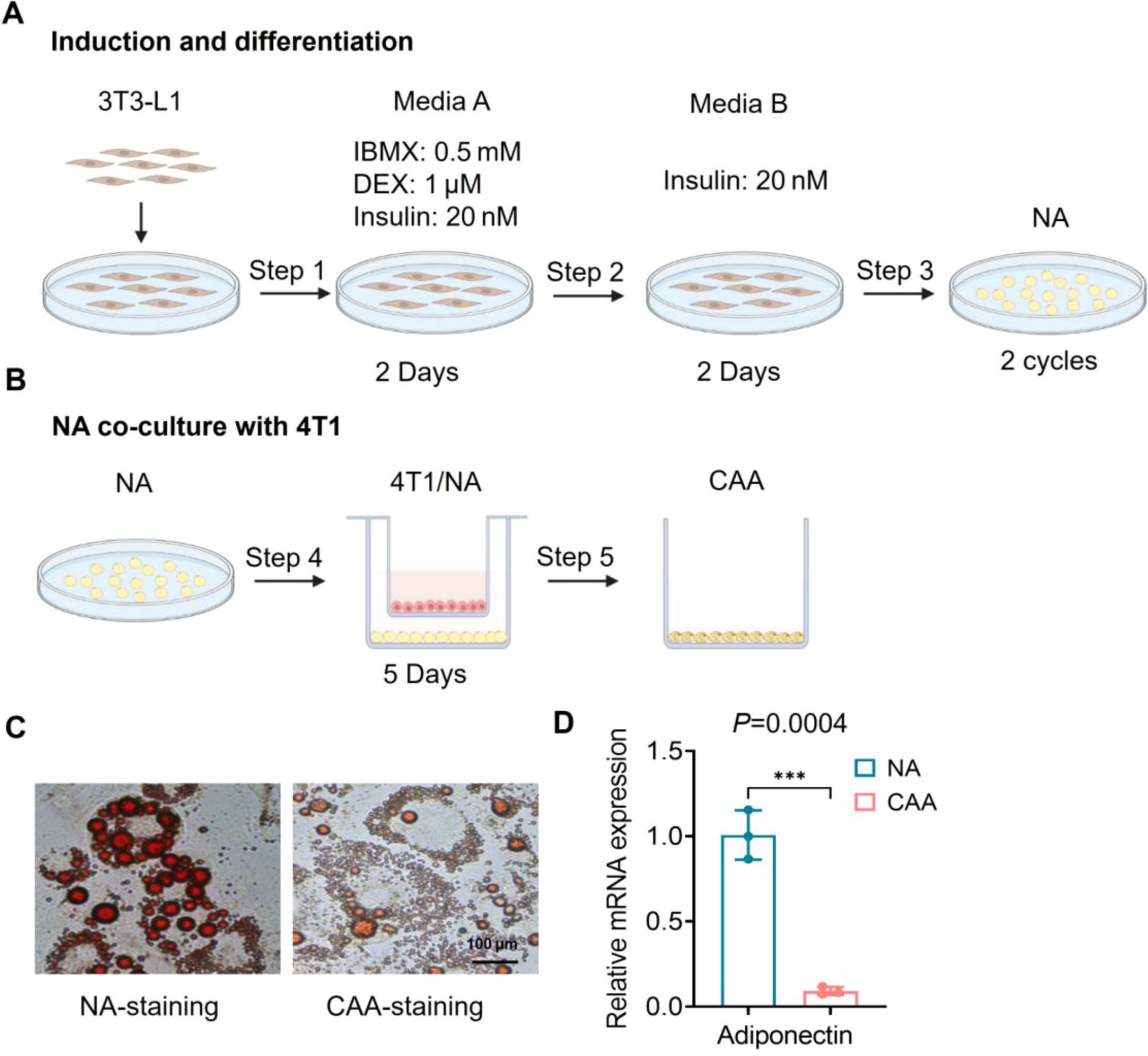
*In vitro* induction and characterization of CAA. **(A)** Differentiation scheme of 3T3-L1 preadipocytes into normal adipocytes (NA). **(B)** Transwell (0.4 μm) non-contact co-culture of NA (lower well) with 4T1 cells (upper insert) for 5 days to generate CAA. **(C)** Oil Red O staining revealed the morphology in NA and CAA. **(D)** RT- qPCR was used to measure the relative mRNA expression levels of adiponectin in adipocytes. Data are presented as mean ± SD; ***P < 0.001

Through the establishment of a Transwell co-culture system comprising adipocytes and 4T1 cells, we successfully generated a model of CAA to mimic tumor-adipose stromal interactions *in vivo* (**Figures 1A, B**). Oil Red O staining revealed that co-cultured adipocytes exhibited a classic CAA phenotype: compared with normal mature adipocytes, they displayed markedly reduced cell size, significantly decreased intracellular lipid accumulation, and fragmented, unevenly distributed lipid droplets (**Figure 1C**). To determine whether adipocytes undergo phenotypic transformation after co-culture with breast cancer cells, we measured the mRNA expression levels of adiponectin in the adipocytes. Adiponectin is a secretory factor specific to mature adipocytes, and its downregulation serves as a key marker of adipocyte dedifferentiation and transformation into cancer-associated adipocytes. The results showed that, compared with adipocytes cultured alone, the mRNA expression levels of adiponectin in adipocytes were significantly downregulated following co-culture with 4T1. These findings suggest that breast cancer cells can induce adipocytes to lose their typical phenotypic characteristics and undergo a transformation into CAA. (**Figure 1D**). Furthermore, under the influence of paracrine signals from TNBC cells, adipocytes undergo functional reprogramming characterized by diminished lipid storage capacity, suggesting a shift toward an energy-supplying and pro-tumor phenotype. The established CAA model effectively recapitulates the pathological features of adipocytes within the TME and provides a reliable *in vitro* platform for further investigation into the role and underlying mechanisms of adipocytes in tumor progression.

### CAA enhances the migratory and invasive capacities of TNBC cells

**Figure 2:**
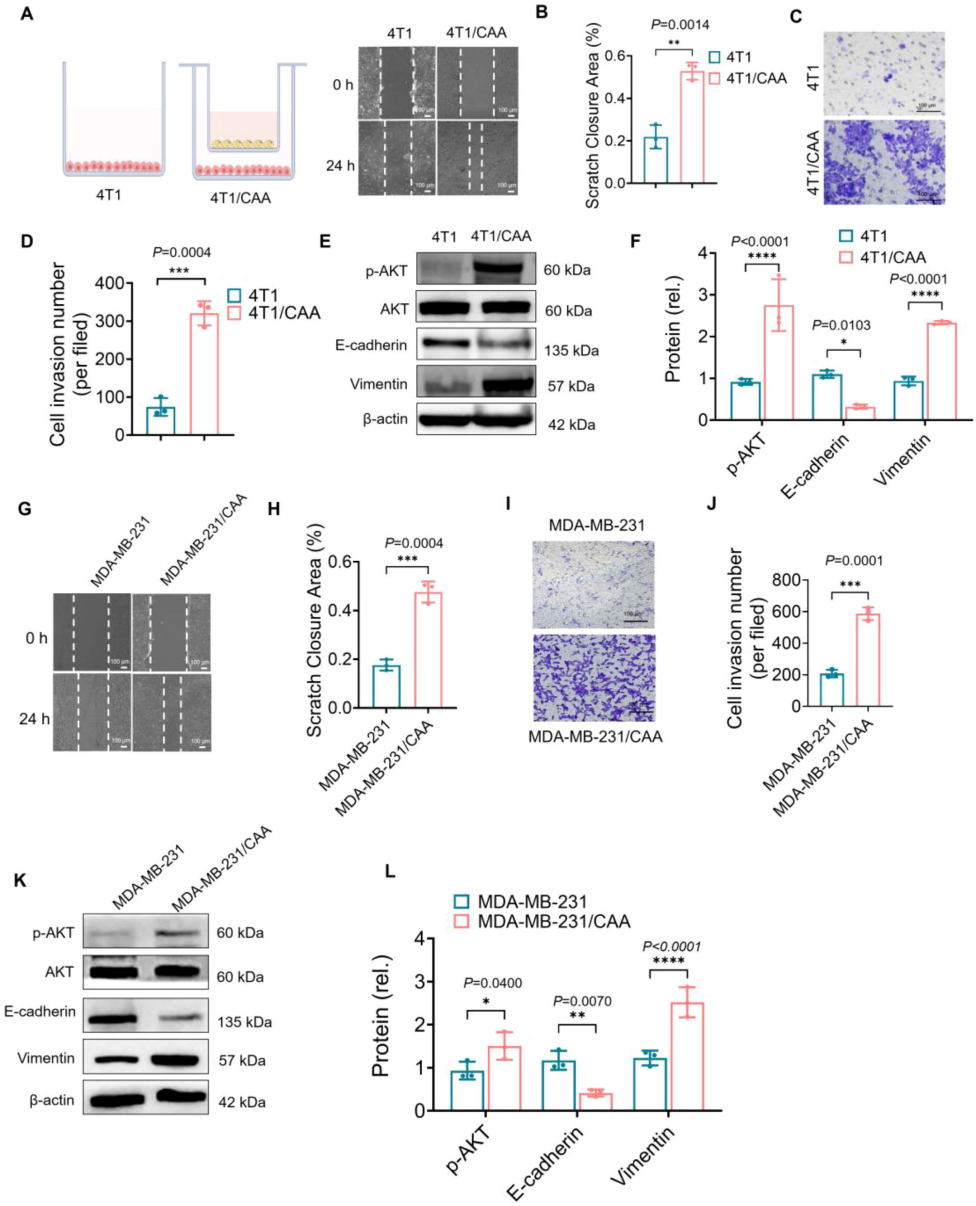
CAA enhances the migratory, invasive and EMT capacities of TNBC cells *in vitro*. **(A, B)** Schematic diagram of the experimental groups: the 4T1-alone group (control) and the CAA co-culture group. Representative images of the wound-healing assay and the corresponding quantitative analysis of wound closure assessed at 0 h and 24 h are shown. **(C, D)** Representative images and statistical analysis of Transwell−Matrigel invasion assays. Invaded cells were stained with crystal violet and counted. **(E, F)** After a 5-day co-culture with CAA, the expression of proteins associated with EMT and the PI3K/AKT signaling pathway was analyzed in 4T1 cells. **(G, H)** Quantitative analysis of the migration capacity and wound healing rate of MDA-MB-231 cells under different treatments using the scratch assay. **(I, J)** Quantitative analysis of cell invasion capacity and the number of invading cells using the Transwell invasion assay. **(K, L)** After a 5-day co-culture with CAA, the expression of proteins associated with EMT and the PI3K/AKT signaling pathway was analyzed in 4T1 cells. Data are presented as mean ± SD; *P < 0.05, **P < 0.01, ***P < 0.001, ****P < 0.0001.

To investigate the CAA on the migratory behavior of TNBC cells, a wound healing (scratch) assay was first performed. The experimental scheme is shown in the figure and comprises two groups: the 4T1-alone group (control) and the CAA co-culture group (**Figure 2A**). The results showed that co-culture with CAA significantly accelerated the wound closure rate of 4T1 cells compared to the control. At the 24-hour time point, the migration rate in the CAA co-culture group was approximately twice that of the control group, whereas only minimal closure was observed in the control group (**Figures 2A, B**). These findings suggest that CAA can markedly enhance the horizontal migration ability of TNBC cells.

To further evaluate the effect of CAA on the invasive capacity of tumor cells, a Transwell-Matrigel invasion assay was subsequently conducted. The experimental groups were set up consistently with the migration assay, including the control group and the CAA co-culture group. The results demonstrated that, consistent with the migration assay, the number of cells penetrating the Matrigel-coated membrane was significantly higher in the CAA co-culture group than in the control group. Crystal violet staining revealed that the CAA co-culture group exhibited a greater number of transmigrated cells with an extended morphology, while the control group showed relatively sparse and round-shaped cells (**Figures 2C, D**). This indicates that CAA can also effectively promote the invasive ability of TNBC cells *in vitro*.

To further investigate the molecular mechanisms underlying CAA-promoted migration and invasion of TNBC cells, we analyzed the expression of proteins associated with EMT and the PI3K/AKT signaling pathway by Western blot. The results showed that, following co-culture with CAA, the expression of the epithelial marker E-cadherin was significantly downregulated in 4T1 cells, while the expression of the mesenchymal markers Vimentin was significantly upregulated. At the same time, the level of phosphorylated AKT (p-AKT) was significantly elevated, whereas total AKT (AKT) remained unchanged (**Figures 2E, F**). These findings suggest that CAA may enhance the motile and invasive capabilities of TNBC cells by activating the PI3K/AKT signaling pathway and inducing the EMT process.

In addition, the same functional assays were performed in MDA-MB-231 cells to validate the generalizability of the above findings. Results from the scratch assay showed that, following co-culture with CAA, the wound healing rate of MDA-MB-231 cells was significantly faster than that of the control group (**Figures 2G, H**). Transwell invasion assays similarly demonstrated that the number of MDA-MB-231 cells penetrating the Matrigel membrane was significantly higher in the CAA co-culture group compared to the control group (**Figures 2I, J**). Western blot analysis further confirmed that, in MDA-MB-231 cells, co-culture with CAA similarly induced downregulation of E-cadherin, upregulation of vimentin, and increased p-AKT levels, while AKT levels remained unchanged (**Figures 2K, L**). These results indicate that the promoting effect of CAA on TNBC cells migration and invasion, as well as its molecular mechanisms, are consistent in both murine and human cells. Collectively, through wound healing, Transwell-Matrigel invasion assays, and molecular mechanistic analyses, this study demonstrated in both 4T1 and MDA-MB-231 cells that CAA not only promotes the migration and invasion of TNBC cells but that its effects are closely associated with the activation of the PI3K/AKT pathway and the induction of EMT. These results systematically reveal the critical role of CAA in driving the malignant phenotype of TNBC *in vitro* and provide direct experimental evidence for the contribution of adipocytes to cancer cell metastasis within the TME.

### The PI3K inhibitor LY294002 reverses CAA-induced migration, invasion, and EMT in TNBC

To investigate the role of the PI3K/AKT signaling pathway in the adipocyte-mediated malignant phenotype of tumor cells, we added the PI3K inhibitor LY294002 to the co-culture system and re-assessed the migration and invasion capabilities of the tumor cells.

**Figure 3:**
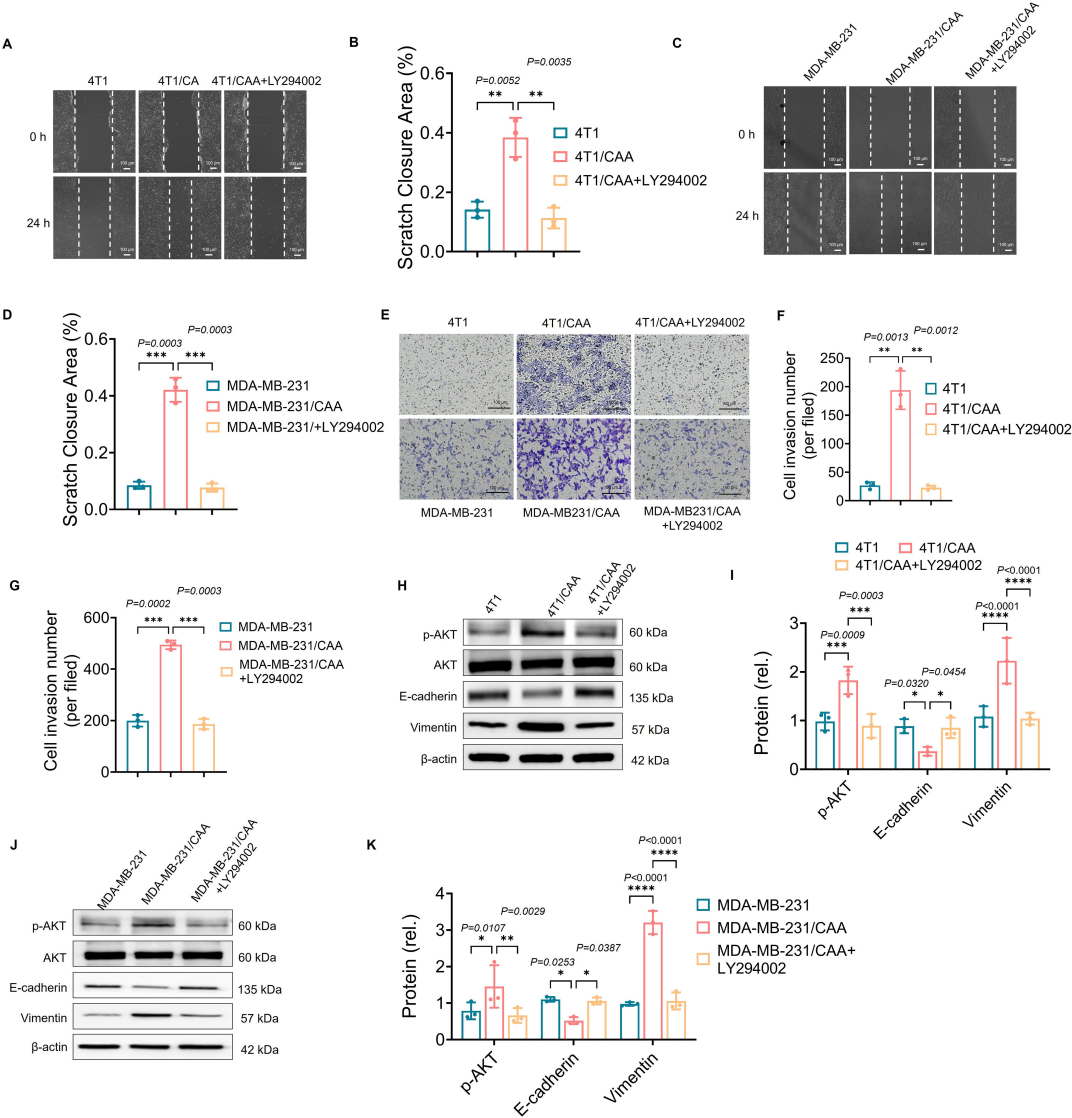
The PI3K inhibitor reverses CAA enhances the migratory, invasive and EMT capacities of cancer cells *in vitro.* **(A, C)** The scratch assay was used to assess the migratory capacity of MDA-MB-231 and 4T1 cells under different treatment conditions. Representative images were captured at 0 h and 24 h. **(B, D)** Quantitative analysis of scratch healing rates. Data are presented as mean ± standard deviation, n = 3. **(E)** Transwell invasion assay to assess the invasive capacity of cells in each group. Crystal violet staining reveals cells that have traversed the matrix gel. **(F, G)** Quantitative analysis of the number of invading cells. Data are presented as mean ± standard deviation, n = 3. **(H, J)** Western blot analysis of protein expression levels of p-AKT, AKT, E-cadherin, and vimentin in MDA-MB-231 and 4T1 cells. **(I, K)** Statistical analysis of grayscale values for relative protein expression levels. Data are presented as mean ± standard deviation, n = 3. Data are presented as mean ± SD; *P < 0.05 **P < 0.01, ***P < 0.001, ****P < 0.0001.

The results of the scratch assay showed that, compared with the control group cultured in isolation, the scratch healing rate was significantly accelerated in MDA-MB-231 and 4T1 cells following co-culture with adipocytes. However, upon treatment with LY294002, the enhanced cell migration capacity induced by co-culture was significantly inhibited, and the healing rate even dropped to a level close to that of the control group (**Figures 3A–D**). The Transwell invasion assay revealed a similar trend. Co-culture significantly enhanced the invasive capacity of both tumor cell lines, whereas treatment with LY294002 effectively blocked this effect, resulting in a significant reduction in the number of invading cells compared to the co-culture-only group (**Figures 3E–G**).

Using Western blot analysis, we further examined changes in EMT-related markers and AKT phosphorylation levels to investigate the molecular mechanism underlying the action of LY294002. The results showed that, compared with the control group, MDA-MB-231 and 4T1 cells exhibited significantly upregulated p-AKT levels after co-culture with adipocytes, accompanied by downregulated E-cadherin expression and upregulated vimentin expression, indicating that co-culture activated the PI3K/AKT pathway and promoted the occurrence of EMT. Upon addition of LY294002, p-AKT expression was significantly inhibited, confirming the efficacy of the inhibitor. More importantly, treatment with LY294002 reversed the co-culture-induced EMT phenotype: E-cadherin expression was restored (upregulated), while the expression of the mesenchymal marker vimentin was significantly downregulated (**Figures 3H–K**). AKT protein expression levels did not show significant changes across groups.

These results suggest that adipocytes induce EMT in TNBC by activating the PI3K/AKT signaling pathway, thereby promoting their migration and invasion capabilities; targeting and inhibiting this pathway can effectively reverse this process.

### CAA promotes *in vivo* tumor growth without inducing host wasting in an orthotopic mammary fat pad model of TNBC

**Figure 4:**
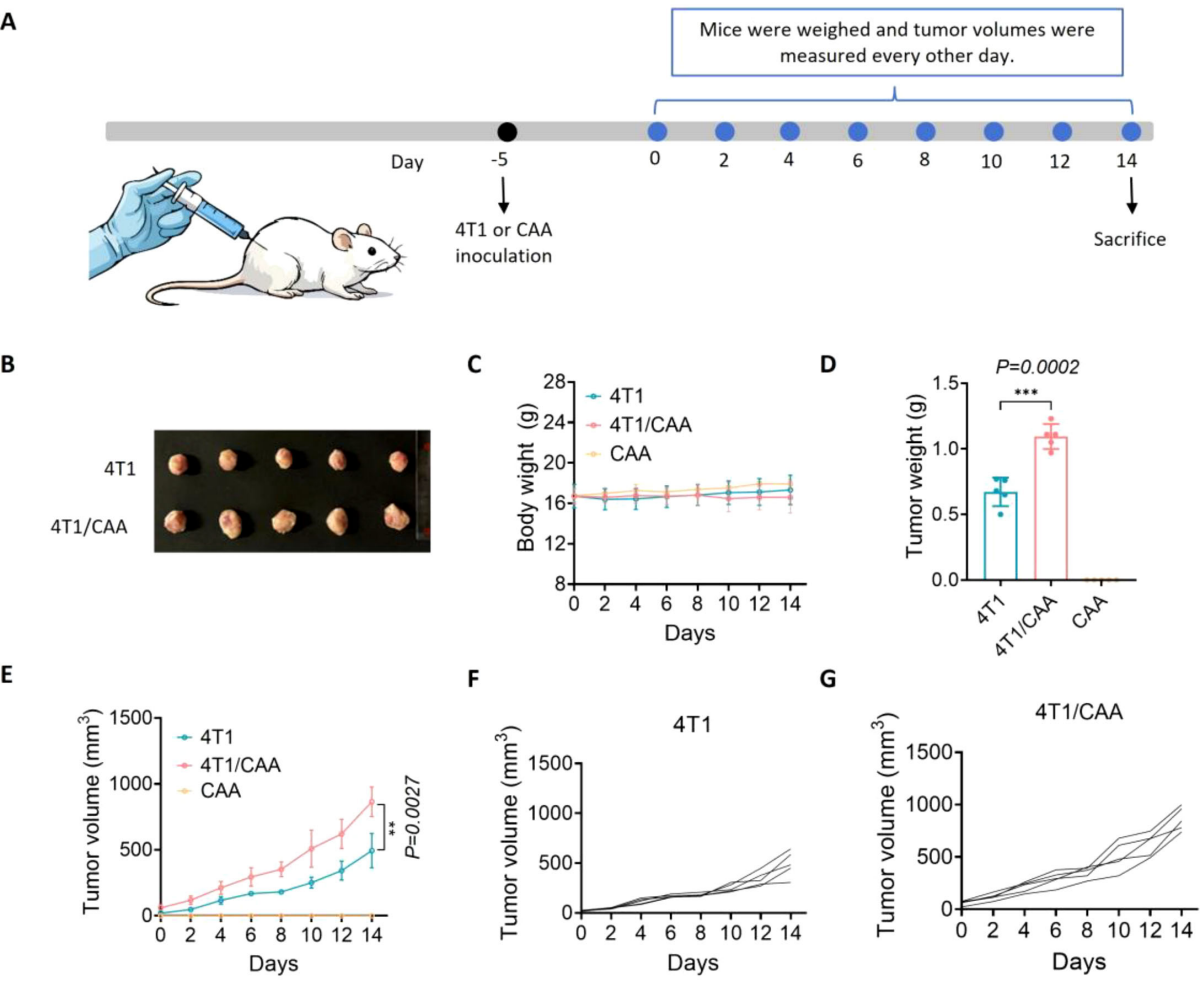
CAA promotes TNBC growth *in vivo*. **(A)** Schematic timeline of orthotopic implantation and monitoring. **(B)** Representative images of orthotopic tumors in the mammary fat pads of BALB/c mice from the 4T1−alone (n=5), 4T1/CAA co−injection (n=5), and CAA−alone (n=5) groups at the experimental endpoint. **(C)** Body weight of mice during the 14-day observation period. **(D)** Tumor weights collected at euthanasia. **(E–G)** Tumor volume growth curves for each group [**(E)** mean; **(F, G)** individual mice]. Data are presented as mean ± SEM; **P < 0.01, ***P < 0.001.

To evaluate the impact of CAA on the *in vivo* progression of TNBC, we established an orthotopic mouse tumor transplantation model. Female BALB/c mice aged 6–8 weeks were randomly divided into three groups: CAA, 4T1cells, and a co-injection group consisting of 4T1 cells mixed with CAA (4T1/CAA). Tumor cells were transplanted via orthotopic injection into the mammary fat pad. The 4T1-alone group received 1×10^6^ 4T1 cells, while the 4T1/CAA co-injection group received a 4:1 mixture of 4T1 cells and CAA. Over a 14-day observation period, tumor growth, body weight change, and overall survival status were systematically monitored (**Figure 4A**).

The results showed that injection of CAA alone had no significant effect on survival indices; all mice remained healthy until the experimental endpoint, with no observable tumor formation or notable body weight loss, confirming that CAA itself is non-tumorigenic under these experimental conditions (**Figures 4B, C**). In contrast, the 4T1/CAA co-injection group exhibited a significant tumor-promoting effect. Both the tumor volume growth rate and the final tumor weight in this group were significantly higher than those in the 4T1-alone group (**Figures 4D–G**). Notably, despite the substantially increased tumor burden, body weight in the 4T1/CAA group showed no statistical difference compared with controls throughout the experiment, indicating that CAA-promoted tumor growth did not induce obvious systemic wasting.

In summary, these *in vivo* findings demonstrate that while CAA lack direct tumor-initiating ability, they can markedly accelerate the growth of TNBC *in vivo* through interactions with tumor cells, without affecting host body weight or baseline survival status. These results further underscore the role of CAA as a key modulator within the TME.

### CAA promotes pulmonary metastasis of TNBC in a mouse model

**Figure 5:**
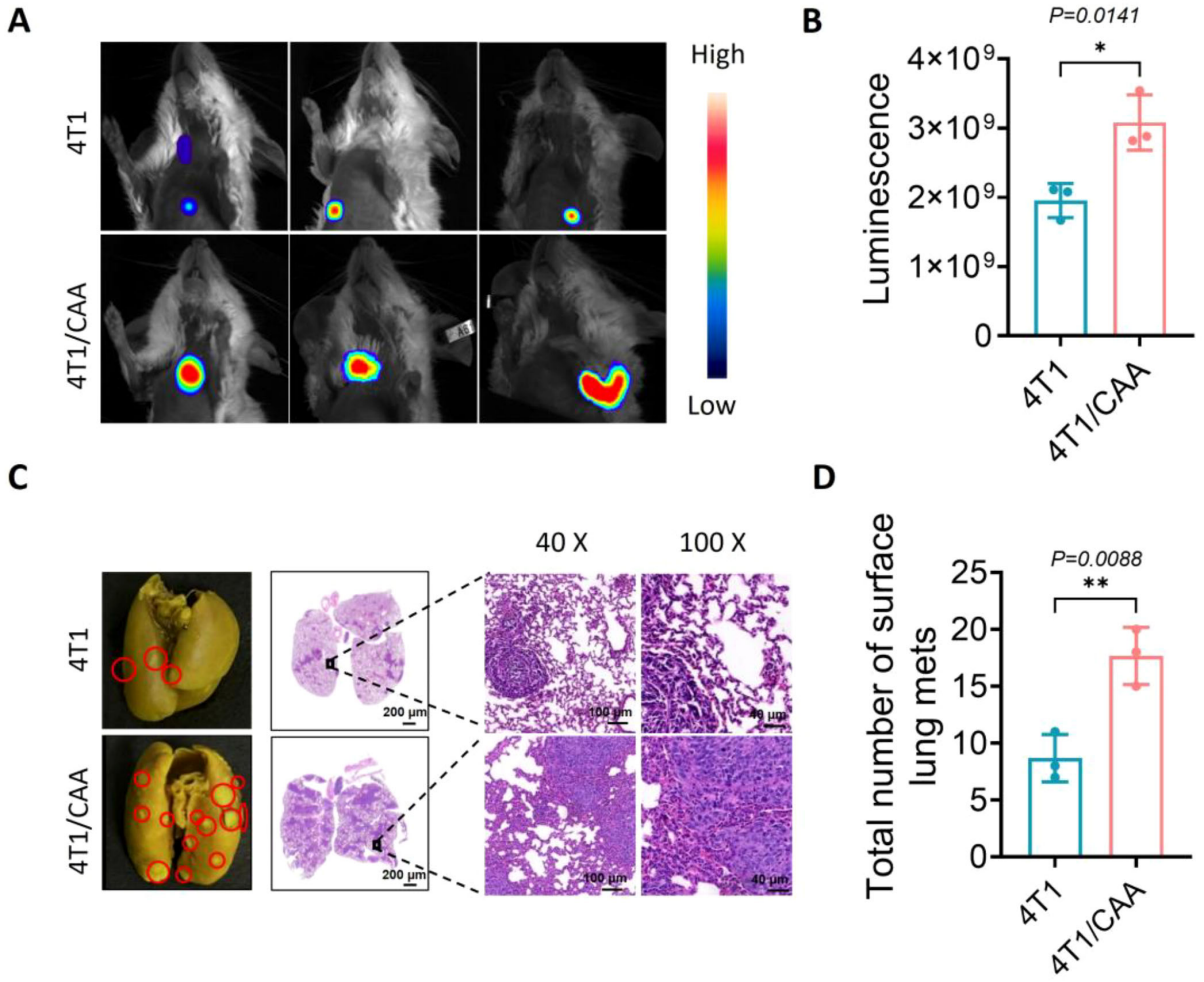
CAA promotes lung metastasis *in vivo*. (**A, B)** Representative bioluminescence images of mice at 35 days post−inoculation. The color scale indicates signal intensity, with red representing the highest bioluminescence signal. **(C, D)** Representative macroscopic images of lung tissues fixed in Bouin’s solution and quantitative analysis of surface metastatic nodules in BALB/c mice from different groups. Metastatic tumor foci are shown by H&E staining. Data are presented as mean ± SEM; *P< 0.05, **P< 0.01.

To further investigate the role of CAA in promoting distant metastasis, we established a lung metastasis mouse model. Systemic analysis via *in vivo* imaging and histopathological examination confirmed that CAA significantly enhances pulmonary metastasis of TNBC. *In vivo* fluorescence imaging revealed that, at day 35 post−inoculation, mice in the 4T1/CAA co−injection group exhibited markedly higher overall signal intensity in the thoracic region compared to the 4T1−alone group, indicating more active dissemination of tumor cells to the lungs (**Figures 5A, B**). Gross examination of lungs after dissection further showed that the number of macroscopic metastatic nodules on the lung surface was significantly increased in the 4T1/CAA group, with nodules being more densely distributed and larger in size. Histological analysis by hematoxylin and eosin (H&E) staining corroborated these findings at the tissue level (**Figures 5C, D**). In contrast to the scattered, microscopic metastatic foci observed in the lungs of the 4T1−alone group, lung tissues from the 4T1/CAA group displayed a substantially greater number of tumor cell clusters, including multiple confluent lesions that extensively infiltrated the pulmonary parenchyma.

Taken together, these results demonstrate that CAA potently facilitates the pulmonary metastatic process of TNBC *in vivo*, further underscoring the critical role of CAA within the metastatic TME.

### CAA promotes TNBC progression through dual mechanisms: enhancing malignant phenotype and fostering an immunosuppressive microenvironment

In an effort to elucidate the mechanisms underlying CAA-mediated tumor progression, we undertook a comprehensive immunofluorescence analysis of orthotopic tumor tissues harvested from mice. The results revealed that tumor tissues from the CAA co-injection group exhibited pronounced malignant phenotypes and features of an immunosuppressive microenvironment compared with the control group.

Regarding tumor-intrinsic characteristics, immunofluorescence staining showed a significant increase in the proportion of Ki67-positive cells, indicating enhanced proliferative activity of tumor cells in the presence of CAA. Concurrently, expression of the epithelial marker E-cadherin was downregulated, suggesting activation of the EMT program. These molecular alterations align with the observed enhancement in migratory and invasive capacities of tumor cells, providing phenotypic support at the molecular level for the role of CAA in promoting malignant progression of TNBC.

**Figure 6:**
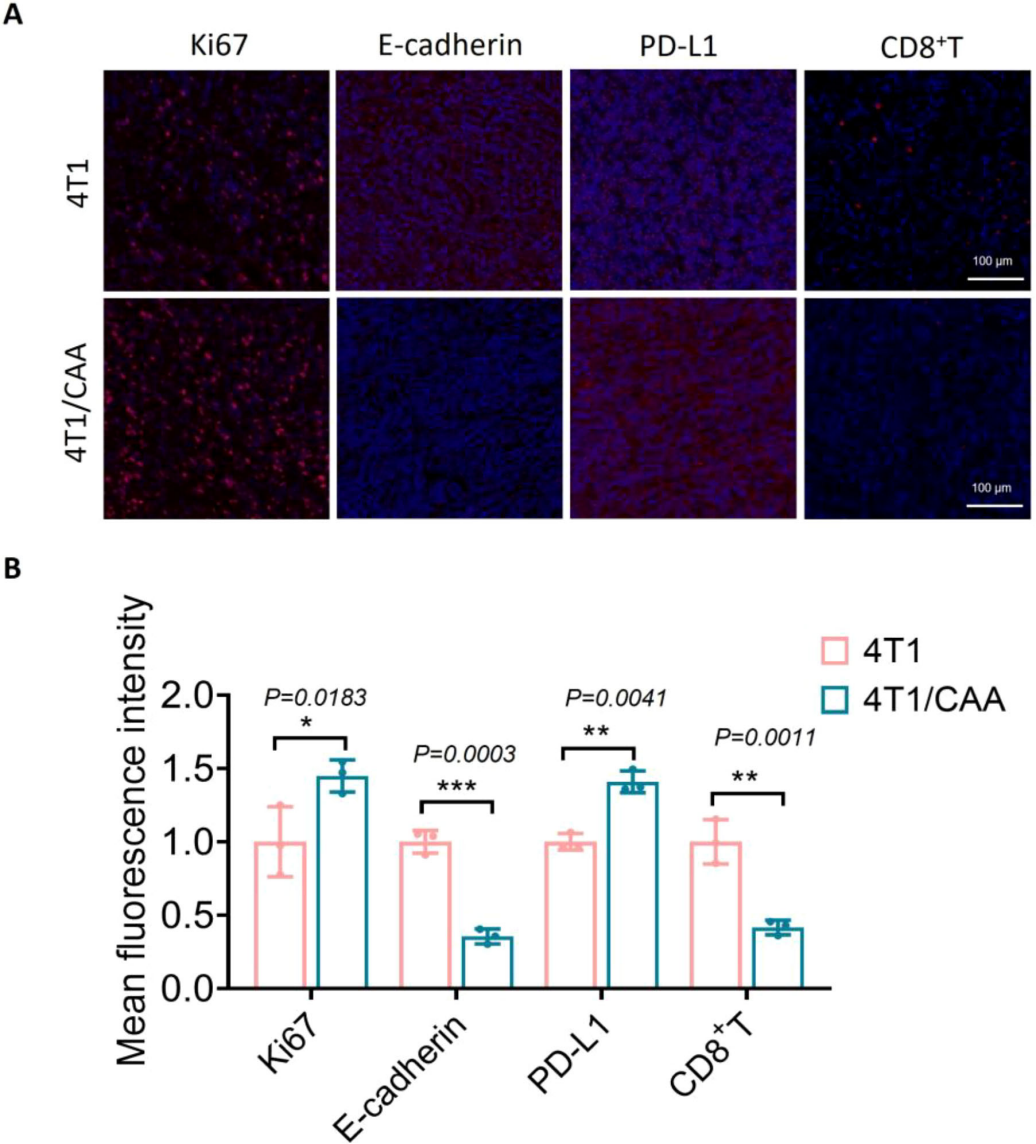
CAA promotes TNBC progression by fostering a protumorigenic phenotype and an immunosuppressive microenvironment. **(A)** Representative immunofluorescence images of Ki67, E-cadherin, PD-L1, and CD8^+^ T cells in tumors from the 4T1 and 4T1/CAA groups. **(B)** Quantification of mean fluorescence intensity. Data are presented as mean ± SEM. *P < 0.05, **P < 0.01, ***P < 0.001.

In terms of the tumor immune microenvironment, analysis revealed a marked upregulation of the immune checkpoint protein PD-L1 on tumor cells. Correspondingly, the infiltration of CD8^+^ T cells into the tumor parenchyma was significantly reduced. Together, these findings suggest that CAA may help foster an immunosuppressive tumor microenvironment by simultaneously upregulating PD-L1 on tumor cells and impairing the infiltration of cytotoxic CD8^+^ T cells (**Figures 6A, B**).

In summary, Immunofluorescence analysis demonstrates that CAA promotes TNBC progression and immune evasion by simultaneously enhancing tumor cell proliferation, activating EMT, and remodeling the tumor immune microenvironment to suppress host anti-tumor immunity.

## Discussion

Adipocytes are one of the most abundant stromal cells in the breast (21). Different from normal adipocytes, the adipocytes adjacent to triple negative breast cancer were remodeled into CAA, showing smaller volume, increased lipolysis, and changes in secretion spectrum, which had a more significant tumor promoting effect (22, 23).They enhance tumor invasion via inflammatory cytokines and matrix-remodeling factors, supply free fatty acids to fuel cancer metabolism, and foster an immunosuppressive environment by recruiting M2 macrophages and regulatory T cells. These pro-metastatic and immune-regulatory functions represent acquired, tumor-induced traits unique to CAA (13). Consistent with these concepts, our co−culture system recapitulated key CAA−like features, including diminished lipid accumulation, supporting the feasibility of using this model to interrogate adipocyte reprogramming. These findings align with clinical observations linking adipocyte-rich microenvironments to aggressive disease and poorer outcomes in TNBC (24).

Furthermore, we demonstrated that CAA significantly enhances the migratory and invasive abilities of TNBC cells and promotes both tumor growth and pulmonary metastasis *in vivo*. These results not only align with previous reports on the tumor−promoting role of CAA in other breast cancer subtypes, but also extend this function explicitly to the more aggressive TNBC context (25, 26). Importantly, unlike earlier studies that primarily focused on the metabolic support provided by CAA (27), our work reveals a dual mechanism through which CAA drives TNBC progression: by modulating intrinsic tumor−cell properties, evidenced by the downregulation of E−cadherin, upregulation of vimentin, and upregulation of p-AKT, suggesting that it promotes EMT and activates the PI3K/AKT pathway; on the other hand, CAA induces an immunosuppressive tumor microenvironment, characterized by elevated PD-L1 expression and reduced CD8^+^ T-cell infiltration, thereby modulating the intrinsic properties of tumor cells. At the tumor−cell−intrinsic level, tumors formed in the presence of CAA exhibited reduced E−cadherin and increased Ki−67, accompanied by activation of the PI3K/AKT pathway. This pattern is consistent with the engagement of EMT−associated programs and proliferative signaling that can facilitate invasion and metastatic dissemination. Previous work suggests that adipocyte−derived cytokines, lipids, and extracellular vesicles can activate oncogenic pathways in cancer cells, including PI3K/AKT and related networks (28, 29). Our data extend these observations by linking CAA exposure to PI3K/AKT activation together with EMT−like marker changes in an orthotopic TNBC context.

Notably, our study also implicates CAA in remodeling the local immune landscape. Tumors from the 4T1/CAA group displayed elevated PD−L1 expression and reduced CD8^+^ T cell infiltration, suggesting attenuation of cytotoxic immune surveillance (30). Mechanistically, PI3K/AKT signaling has been reported to regulate PD−L1 expression in several tumor types, providing a plausible node through which CAA−derived cues could couple metabolic/proliferative signaling to immune escape. In addition, CAA may contribute to immune suppression through paracrine inflammatory mediators, altered lipid availability, or the recruitment/education of suppressive myeloid populations, collectively positioning CAA as a hub linking tumor metabolism with immune evasion (31–33).

This study has several limitations. First, we used the Transwell assay to capture paracrine communication, which primarily allows for the specific analysis of paracrine factor effects but does not replicate the full complexity of direct cell-cell contact, extracellular matrix remodeling, or the lipid-rich TME. Second, this study employed a PI3K inhibitor to validate the necessity of this pathway; however, other effector molecules may exist downstream of PI3K. Future studies will further utilize specific AKT inhibitors or Akt siRNA knockdown experiments to precisely define the specific role of AKT in this process. Finally, our immune characterization was limited to PD−L1 and CD8^+^ T cells; future studies should profile additional immune subsets and functional readouts (e.g., cytokine production, exhaustion markers) and identify the specific adipocyte-derived factors responsible. Addressing these points may also inform rational combination strategies that target CAA-tumor crosstalk to enhance the efficacy of chemotherapy and immunotherapy.

In summary, this study systematically elucidates the multifaceted role of CAA in the progression of TNBC. CAA not only directly enhances the invasive capacity of tumor cells by inducing EMT but also fosters an immunosuppressive microenvironment through upregulation of PD-L1 and suppression of cytotoxic T-cell infiltration, thereby holistically promoting tumor growth and metastasis. These findings deepen our understanding of tumor-adipocyte crosstalk and suggest that targeting CAA or the molecular pathways they mediate may represent a novel strategy to improve therapeutic outcomes in TNBC. Future translational efforts should focus on turning CAA from tumor “accomplices” into actionable therapeutic targets.

## Data Availability

The original contributions presented in the study are included in the article/supplementary material. Further inquiries can be directed to the corresponding authors.
