# Correction: Cancer-associated adipocyte promote progression and immunosuppression in triple-negative breast cancer

**DOI:** 10.3389/fonc.2026.1858466

**Published:** 2026-05-05

**Authors:** Xichao Zhang, Shenjie Zhong, Shan Liu, Xiaofeng Mu, Gang Chen, Wen Chen

**Affiliations:** 1 School of Medical Laboratory, Shandong Second Medical University, Weifang, China; 2 Department of Clinical Laboratory, Qingdao Central Hospital, University of Health and Rehabilitation Sciences, Qingdao, China; 3 School of Rehabilitation Science and Engineering, University of Health and Rehabilitation Sciences, Qingdao, China; 4 Emergency Center, Qingdao Central Hospital, University of Health and Rehabilitation Sciences, Qingdao, China

**Keywords:** cancer-associated adipocyte, epithelial-mesenchymal, transition, immunosuppressive microenvironment, triple-negative breast cancer, tumor microenvironment

Author Wen Chen was erroneously assigned to affiliation “Department of Clinical Laboratory, Qingdao Central Hospital, University of Health and Rehabilitation Sciences, Qingdao, China”. This affiliation has now been removed for author Wen Chen.

Affiliation “Emergency Center, Qingdao Central Hospital, University of Health and Rehabilitation Sciences, Qingdao, China” was omitted for author Wen Chen. This affiliation has now been added for author Wen Chen.

The original version of this article has been updated.

